# A Novel Rapid Detection Method for *Mycobacterium tuberculosis* Based on Scattering-Light Turbidity Using Loop-Mediated Isothermal Amplification

**DOI:** 10.3390/bios15030162

**Published:** 2025-03-03

**Authors:** Meimei Zeng, Xinru Wang, Zifeng Tan, Wenyan Guo, Yan Deng, Song Li, Libo Nie, Nongyue He, Zhu Chen

**Affiliations:** 1 Hunan Key Laboratory of Biomedical Nanomaterials and Devices, Hunan University of Technology, Zhuzhou 412007, China; mmzeng628@163.com (M.Z.); 18686704507@163.com (X.W.); tzf2601031469@163.com (Z.T.); gwyan3410@hut.edu.cn (W.G.); nyhe@seu.edu.cn (N.H.); 2 MOE Key Lab of Rare Pediatric Diseases, Hengyang Medical College, University of South China, Hengyang 421001, China; hndengyan@126.com (Y.D.); sosong1980@gmail.com (S.L.); 3 Institute for Future Sciences, University of South China, Changsha 410008, China; 4 State Key Laboratory of Digital Medical Engineering, School of Biological Science and Medical Engineering, Southeast University, Nanjing 210096, China

**Keywords:** *Mycobacterium tuberculosis*, loop-mediated isothermal amplification, turbidimetry, scattering method

## Abstract

The accurate detection of *Mycobacterium tuberculosis* (MTB) is a pressing challenge in the precise prevention and control of tuberculosis. Currently, the efficiency and accuracy of drug resistance detection for MTB are low, and cross-contamination is common, making it inadequate for clinical needs. This study developed a rapid nucleic acid detection method for MTB based on scattering loop-mediated isothermal amplification (LAMP). Specific primers for the MTB-specific gene (*Ag85B*) were designed, and the LAMP reaction system was optimized using a self-developed scattering LAMP turbidimeter. Experimental results showed that the optimal reaction system included 1.5 µL of 100 mmol/L magnesium ions, 3.5 µL of 10 mmol/L dNTPs, 6 µL of 1.6 mol/L betaine, and a reaction temperature of 65 °C. The minimum detection limit was 12.40 ng/L, with the fastest detection time being approximately 10 min. The reaction exhibited good specificity, with no amplification bands for other pathogens. Twenty culture-positive samples and twenty culture-negative samples were tested in parallel; the accuracy of the positive group was 100%, the detection time was (24.9 ± 13 min), and there was no negative detection. This method features high detection efficiency, low cost, high accuracy, and effectively reduces cross-contamination, providing a new technology for the rapid clinical detection of MTB.

## 1. Introduction

Tuberculosis (TB) is a chronic respiratory infectious disease caused by *Mycobacterium tuberculosis* (MTB), posing a serious threat to human health [1]. In 2023, the global number of new TB cases was 10.8 million (95% confidence interval: 10.1 million–11.7 million), showing a slight increase compared to 10.7 million in 2022 but still significantly higher than the 10.4 million cases in 2021 and 10.1 million cases in 2020. The reduction in TB-related deaths and the slowing of the incidence rate increase indicate that TB diagnosis and treatment efforts have significantly recovered since the COVID-19 pandemic. Despite progress in combating TB, it may have re-emerged as the leading cause of death from a single infectious pathogen worldwide [2]. At the 2023 United Nations High-Level Meeting on TB, new global targets were set for 2027, aiming for 100% of newly diagnosed patients to undergo rapid diagnostic tests at the first consultation (compared to 48% in 2023). Early TB diagnosis is important to achieve this goal.

Additionally, drug resistance is a critical issue limiting the prevention and control of TB [3,4,5]. Drug-resistant tuberculosis (DR-TB) significantly contributes to the global burden of antimicrobial resistance [6] and often consumes a substantial portion of healthcare budgets and resources in many endemic countries [7,8]. At the same time, diagnostic centers in low- and middle-income countries may not have access to the most advanced and up-to-date diagnostic technologies, leading to false-negative results and making treatment decisions more challenging. Therefore, there is an urgent need for updated and more accessible diagnostic methods to curb the spread of these resistant strains worldwide [9].

In the development of rapid and reliable TB diagnostic technologies, several antigens have been extensively studied, including *CFP-10*, *ESAT-6*, *Ag85A*, *Ag85B*, *CFP-7*, and PPE18. Some of these have been identified as biomarkers, becoming key elements for assessing the interaction between the pathogen and the host immune response, which is critical for the detection, treatment, and control of tuberculosis [10]. Among them, *Ag85B* shows higher sensitivity and specificity [11]. *Ag85B* is an early secretory protein encoded by the Rv1886c gene of MTB, with strong immunogenicity that can induce both T-cell and B-cell immune responses. Its potential in the early diagnosis of TB has been partially validated, especially in patients with a history of anti-TB treatment, where detection of *Ag85B* expression provides crucial diagnostic information [12]. Dong, Y. et al. emphasized that the detection of *Ag85B* expression is essential for TB diagnosis, particularly in distinguishing between new and recurrent cases, thereby supporting the development of more effective treatment strategies [13].

The screening and diagnosis methods for tuberculosis mainly include microbiology, imaging, immunology, and molecular biology techniques. In microbiological diagnosis, smear microscopy is widely used in clinical practice, but its sensitivity is relatively low and is limited by the number of *Mycobacterium tuberculosis* in sputum. *Mycobacterium tuberculosis* culture is still the gold standard for the diagnosis of tuberculosis; although its sensitivity has improved in recent years, the detection cycle of this method is long (2 to 8 weeks), which not only delays treatment but also significantly increases the risk of pathogen transmission; imaging-based tuberculosis diagnosis methods mainly rely on X-ray examinations and CT scans, especially in the screening and evaluation of pulmonary tuberculosis [14]. Chest X-ray examination is low-cost, simple to operate, and widely used for screening of tuberculosis. However, this method is not sensitive to early tuberculosis, mild tuberculosis, or asymptomatic latent tuberculosis. The sensitivity of CT is higher than that of X-ray, but it may still not detect the early stages of tuberculosis. It is also expensive and may expose patients to radiation damage. Therefore, imaging examinations usually need to be combined with other clinical testing methods to make a confirmed diagnosis; common immunological methods include the tuberculosis skin test (TST), the interferon-γ release assay (IGRA), antibody and antigen detection, immunohistochemical staining, etc. Skin tests are simple to perform and low cost, making them suitable for large-scale screening in resource-limited areas. However, they may produce false positives in individuals with low immunity or those who have been vaccinated with the bcg vaccine (BCG). The interferon-γ release test is a blood test that assesses the response of an individual’s immune system to specific antigens of *Mycobacterium tuberculosis*. Like skin tests, it cannot determine whether the patient has active or latent tuberculosis [15]. Immunological tuberculosis diagnostic methods are often used to assess the immune response to *Mycobacterium tuberculosis* infection, especially in the screening of latent tuberculosis infection. In practical applications, these immunological detection methods are often combined with other imaging or microbiological methods to ensure the accurate diagnosis of tuberculosis. Compared with the traditional combination of microbiology, immunology, and imaging, molecular biology-based nucleic acid detection methods have higher certainty, specificity, and controllability, and are very important means in the diagnosis of modern tuberculosis, especially in the detection of active tuberculosis and drug-resistant tuberculosis [16,17].

A commonly used nucleic acid-based diagnostic method is polymerase chain reaction (PCR). Due to its high sensitivity and specificity, PCR has become the gold standard in the field of molecular diagnostics. However, PCR-based methods are complex to operate, time-consuming, labor-intensive, and require skilled personnel. Additionally, they rely on expensive, large equipment for temperature regulation and signal analysis, which limits their application in certain diagnostic settings [18]. Loop-mediated isothermal amplification (LAMP) is a nucleic acid amplification technology published by Notomi et al. in 2000 [19]. Four to six specific primers are designed for the six regions of the target gene. Under the action of strand displacement DNA polymerase (Bst DNA polymerase), isothermal amplification can be performed in the temperature range of 60–65 °C. The LAMP technology process is roughly divided into five steps, namely nucleic acid extraction, primer design, the LAMP reaction, result detection, and reaction optimization. The sensitivity of LAMP technology is closely related to the quality of nucleic acid extraction. Therefore, nucleic acid extraction is a key step. Nucleic acid extraction can be performed using commercial kits or manual extraction methods. The LAMP reaction requires at least four specific primers, namely outer primers (F3 and B3) and inner primers (FIP and BIP). These four primers form multiple circular amplifications by recognizing different regions of the target DNA. The primer design of LAMP is extremely complex and can be designed using primer design software dedicated to LAMP. The LAMP reaction can be divided into three stages: replication initiation, cyclic amplification, and extension. In the replication initiation stage, since the double-stranded DNA is in a dynamic equilibrium stable state at 65°C, a LAMP primer can bind to the complementary sequence of the double-stranded target gene, and under the action of the strand displacement DNA polymerase, the synthesis of DNA is initiated, and finally a dumbbell-shaped complementary chain is formed, which is the starting material for entering the cyclic amplification. Then, through continuous strand displacement, it enters the cyclic amplification stage, and finally the extension stage. The final product of the amplification is a mixture of DNA with different stem-loop structures and multi-ring cauliflower-like structures, and the product DNA is an alternating reverse repeat sequence of the amplified target sequence [20].

Detection methods for LAMP products primarily include gel electrophoresis, fluorescent dye detection, and turbidity detection [21]. Gel electrophoresis features bright colors and is easy to observe, but its downside is the risk of aerosol contamination when opening the cover. The fluorescent dye method does not require probes and can monitor the amplification of any double-stranded DNA, but its drawbacks include the potential for false-positive signals and high costs [22]. During the DNA synthesis process of the LAMP reaction, dNTPs are consumed to generate pyrophosphate ions, which react with magnesium ions in the reaction system to generate white magnesium pyrophosphate precipitates. Therefore, it is possible to determine whether a precipitate is produced by visual inspection or turbidity detection, thereby evaluating the qualitative results. However, visual inspection has low sensitivity and is prone to misinterpretation, which can lead to experimental bias. Current visual detection methods exhibit poor linearity at low concentrations [23,24]. Turbidimetry and nephelometry are both methods that measure the interaction between light and particles to determine the concentration of particles in a suspension or colloidal solution. Turbidimetry measures the intensity of transmitted light and compares it with the intensity of incident light to estimate the concentration of particles in the solution. Turbidimetry is not sensitive to the precipitation of smaller particles, is greatly affected by sample clarity, particle size, and shape, and is easily disturbed by background light. Nephelometry, also known as scattering turbidimetry, measures the intensity of scattered light at a specific angle (typically 90°) to determine the concentration of particles in the solution. Scattering turbidimetry has the advantage of higher sensitivity, especially for detecting low concentrations of particulate matter [25], and is widely used for measuring substances that can form suspensions.

Finally, in order to improve the sensitivity and specificity of the LAMP reaction, the reaction conditions (such as reaction temperature, magnesium ion concentration, etc.) may need to be optimized. Compared to traditional PCR, LAMP has several advantages, including simpler operation, shorter reaction times, and higher specificity and sensitivity in complex genomic backgrounds, making it a more effective alternative for the rapid and accurate diagnosis of tuberculosis. LAMP reactions do not require high-temperature conditions, which saves on equipment costs, making the method suitable for use in grassroots laboratories and field environments, thus effectively reducing the disease burden in low- and middle-income countries. Wang, R. et al. utilized miRNAs as loop primers in the LAMP reaction, achieving ultra-sensitive and rapid detection, providing a fast, specific, and efficient method for detecting target miRNAs [26]. Wu, Y. et al. developed freeze-dried bead reagents for reverse transcription LAMP (RT-LAMP) to detect *Norovirus GII*, combined with a commercial fully automated nucleic acid analyzer based on magnetic bead and fluorescence detection, establishing a closed-box, integrated, room-temperature storage reagent platform for the on-site immediate detection of *Norovirus GII* [27]. Witkowska, M.W. et al. developed a user-friendly, low-cost prototype device based on reverse transcriptase LAMP (RT-LAMP) for the early diagnosis of Hepatitis C virus [28].

**Figure 1:**
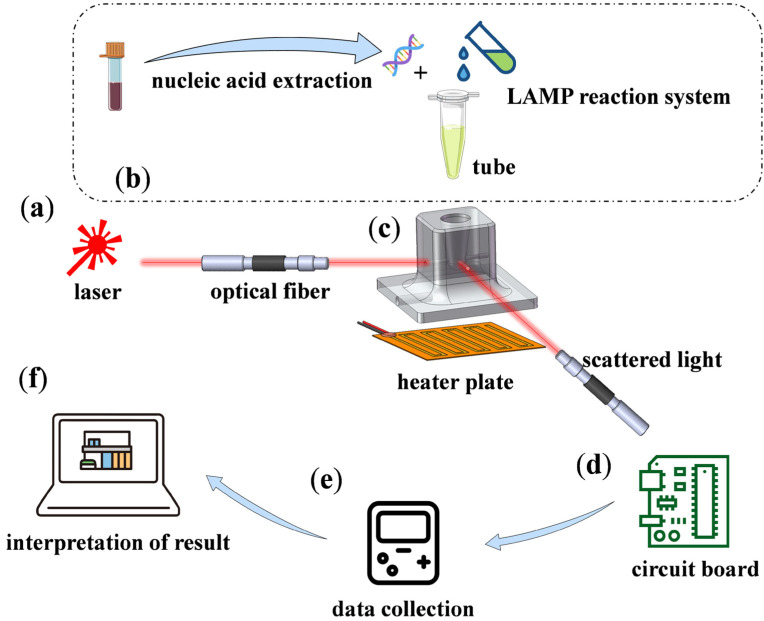
Reaction device setup: (**a**) The laser emits red light with a wavelength of 650 nm and a power of 0.2 W, transmitted through the incident optical fiber to the reaction device; (**b**) MTB samples obtained from the hospital undergo nucleic acid extraction to obtain DNA, which is then mixed with the LAMP reaction system and placed in the reaction well; (**c**) the heating plate is used to raise the temperature of the reaction well to 65 °C, initiating the LAMP reaction; (**d**) the output optical fiber transmits scattered light from the LAMP reaction system to the photodetector on the circuit board, where the signal is filtered and amplified; (**e**) the data acquisition card collects the voltage signal; (**f**) the computer collects the turbidity data for analysis. The data are plotted using Origin to obtain the reaction results.

We have developed a reaction device, as shown in Figure 1. Above the heating device, there is a reaction well for placing PCR tubes. The device uses a laser with a wavelength of 650 nm and a power of 0.2 W to emit incident light, which is transmitted to the bottom of the reaction well via optical fibers. MTB samples obtained from hospitals undergo nucleic acid extraction to obtain genomic DNA. During the experiment, the extracted DNA is mixed with the LAMP reaction system and added to the PCR tube. Below the heating device, an optical fiber hole is reserved to fix the optical fiber position, ensuring that the incident light enters horizontally and the scattered light exits at a 90° angle to the incident light. A polyimide heating film is attached to the bottom of the heating device for heating. Polyimide heating films are widely used in heating applications due to their lightweight, high temperature control precision, and other advantages. A 3D model of the heating device was designed using SolidWorks.2024.SP5.0 and processed accordingly. The photodetector on the circuit board receives the refracted light passing through the LAMP reaction system and amplifies it. The amplified signal is then collected by a data acquisition card, and the final experimental results are displayed on a computer. This device is low-cost, compact, and portable, making it a practical solution to support the development of faster detection methods.

Thus, the aim of this project is to establish a rapid detection method for LAMP products based on scattering turbidimetry, providing a basis for better prevention and treatment of MTB-related diseases.

## 2. Materials and Methods

### 2.1. Reagents and Equipment

A TlANpure Mini Plasmid Kit (DP104) was purchased from Tiangen Biotech Ltd. (Beijing, China). A *Mycobacterium tuberculosis* nucleic acid detection kit (PCR-fluorescent probe method) was purchased from Geju Medical Technology Co., Ltd. (Hangzhou, China). A Mag-MK Bacterial Genomic DNA Extraction Kit (B518725-0050), 2X Lamp Master Mix (Universal), Agarose Regular, Ethidium bromide, 6× DNA Loading Buffer, betaine, DNA marker (500–15,000 bp), and 1× TAE Buffer were obtained from Sangon Biotech. (Shanghai, China). Plasmids of MTB were prepared by Gene-optimal Science & Technology Co., Ltd. (Shanghai, China). *Pseudomonas aeruginosa*, *Escherichia coli*, *Helicobacter pylori*, *Staphylococcus aureus*, and *Mycobacterium tuberculosis* culture positive bacterial solutions and culture negative bacterial solutions were obtained from Zhuzhou Central Hospital (Zhuzhou, China).

The main instruments used in this experiment were DYY-10C nucleic electrophoresis apparatus (Beijing Liuyi Biotechnology Co., Ltd., Beijing, China ), an Applied Biosystems 7500/7500 Fast Real-Time PCR System (Thermo Fisher Scientific, Waltham, MA, USA), and an ND-100C Ultra-micro Ultraviolet Visible Spectrometer (Hangzhou MIU Instruments Co., Ltd., Hangzhou, China). The scattering turbidimeter was self-manufactured by our laboratory [29].

### 2.2. Preparation of DNA Templates

Plasmids of MTB were transformed into *Escherichia coli*, followed by overnight culture at 37 °C. Single colonies exhibiting robust growth were picked and cultured with shaking for 12–16 h. Genomic DNA extraction from bacterial cultures was performed according to the instructions of the TlANpure Mini Plasmid Kit. The extracted plasmids were quantified and assessed for purity using a microvolume UV–Vis spectrophotometer. Several additional bacteria were extracted using a Mag-MK Bacterial Genomic DNA Extraction kit according to the instructions.

### 2.3. LAMP Assays

#### 2.3.1. Design of Primer for LAMP Assays

Based on the published *Ag85B* gene sequence of MTB in GenBank (AB716955.1), Three pairs of primers were selected for six different regions of the gene using the NEB LAMP primer design tool (version 1.4.2.). Each set includes internal primers (FIP and BIP), external primers (F3 and B3), and loop primers (LF and LB). The sequence information is presented in Table 1.

#### 2.3.2. Reaction System and Turbidity Measurement

The extracted DNA solution was thawed from a −20 °C freezer and vortexed to mix thoroughly, followed by centrifugation for 30 s. Primers, Bst DNA polymerase, LAMP Buffer, dNTPs, a magnesium ion solution, and a betaine solution were mixed using a vortex mixer. After preparation, 2.5 µL of LAMP Buffer, 2 µL of DNA, 2 µL of primers, and 1 µL of Bst DNA polymerase were added to PCR tubes using a pipette, maintaining consistent template and varying a single variable for testing, with ddH_2_O added to make the final volume 25 µL. Mineral oil (30 µL) was added to each tube to prevent evaporation. The prepared samples were thoroughly mixed and centrifuged to ensure homogeneity. Real-time turbidity was monitored using a self-developed device. After completion of the experiment, PCR tubes were centrifuged for 1 min in a handheld centrifuge, and successful amplification was assessed visually by the presence of white magnesium pyrophosphate precipitate. Gel electrophoresis was performed subsequently to verify the experimental results.

### 2.4. Optimization of Reaction System

A reaction system containing 2.5 µL of LAMP Buffer, 1.0 µL of Bst DNA polymerase, 2 µL of DNA template, and ddH_2_O added to make a total volume of 25 µL was used. Optimization was performed for reaction conditions including temperature, magnesium ions (concentration of 100 mmol/L), dNTPs (concentration of 10 mmol/L), and betaine (concentration of 1.6 mol/L) volumes (Table 2). Real-time turbidity measurement was conducted using a LAMP turbidimeter, and results were further validated by gel electrophoresis. Data analysis was conducted to determine the optimal reaction system.

### 2.5. Specificity Assays

Using the optimized reaction conditions and system, a DNA template of MTB was used as a positive control, while *Pseudomonas aeruginosa*, *Escherichia coli*, *Helicobacter pylori*, *Staphylococcus aureus*, and sterile water were used to verify the specificity of the LAMP reaction. The results were verified according to the presence or absence of step-like bands in agarose gel electrophoresis.

### 2.6. Sensitivity Assays

An appropriate amount of extracted template DNA was used, with a measured concentration of 12.40 mg/L and an A260/A280 ratio of 1.87. Starting from a template DNA concentration of 12.40 mg/L, serial dilutions were prepared in 10-fold gradients, resulting in concentrations of 12.40 mg/L, 1.24 mg/L, 124.00 µg/L, 12.40 µg/L, 1.24 µg/L, 124.00 ng/L, 12.40 ng/L, and 1.24 ng/L. The template DNA gradient dilutions were added to the optimized reaction conditions for LAMP amplification. The cycle parameters were set according to the instructions of the kit, and the Mycobacterium tuberculosis nucleic acid detection kit (PCR-fluorescent probe method) was used to detect DNA samples of 12.4 mg/L-1.2 µg/L. We also performed statistical analysis on the results of the reaction. Perform statistical analysis on the results of the reaction.

### 2.7. Testing of MTB

Genomic DNA was extracted from culture-positive and culture-negative M. tuberculosis bacterial solutions using a Mag-MK Bacterial Genomic DNA Extraction Kit, and each bacterial solution was extracted 20 times according to the manufacturer’s instructions to obtain 20 MTB-positive and 20 MTB-negative samples. DNA purity was determined using a UV–visible differential photometer. The extracted 40 DNA samples were used as templates for detection by the self-developed scattering LAMP turbidimeter. Accuracy and amplification lift time (TTR, Time to Results) were calculated from the obtained assay data. Accuracy = (true positive + true negative) cases/total cases × 100%. The voltage value of the scattering circuit in the first 5 min was used as the baseline, and 10 times the baseline standard deviation was used as the threshold (TH). Within 60 min, if the transmission voltage value exceeded the TH voltage value, the sample was judged to be MTB-positive; otherwise, the sample was MTB-negative. The time experienced by the transmitted voltage of each reaction tube to reach the set threshold was recorded as TTR. The smaller the TTR value, the faster the detection time. The results were statistically analyzed.

## 3. Results

### 3.1. Optimization of the LAMP Reaction System

#### 3.1.1. Reaction Temperature

**Figure 2:**
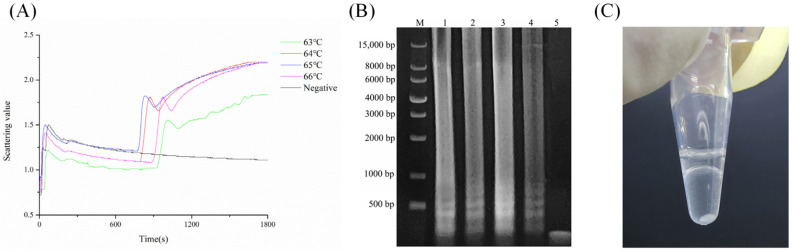
Temperature optimization: (**A**) turbidity scatter plots at different temperatures; (**B**) electrophoresis of amplification products with M-DNA marker: 1—63 °C, 2—64 °C, 3—65 °C, 4—66 °C, 5—negative control; (**C**) precipitate from the 65 °C amplification reaction.

LAMP experiments were conducted at different temperatures. The peak scattering time of the turbidity curve at 65 °C was determined to be 822 s (Figure 2A), which was earlier than at other temperatures. Electrophoresis amplification curves (Figure 2B) showed the brightest band in lane three (65 °C), with lanes one (63 °C), two (64 °C), and four (66 °C) exhibiting dimmer bands and no amplification bands in the negative control. Further visual inspection revealed white precipitates (Figure 2C). Therefore, these results confirm that the optimal reaction temperature for the LAMP reaction is 65 °C.

#### 3.1.2. Magnesium Ion Amount

**Figure 3:**
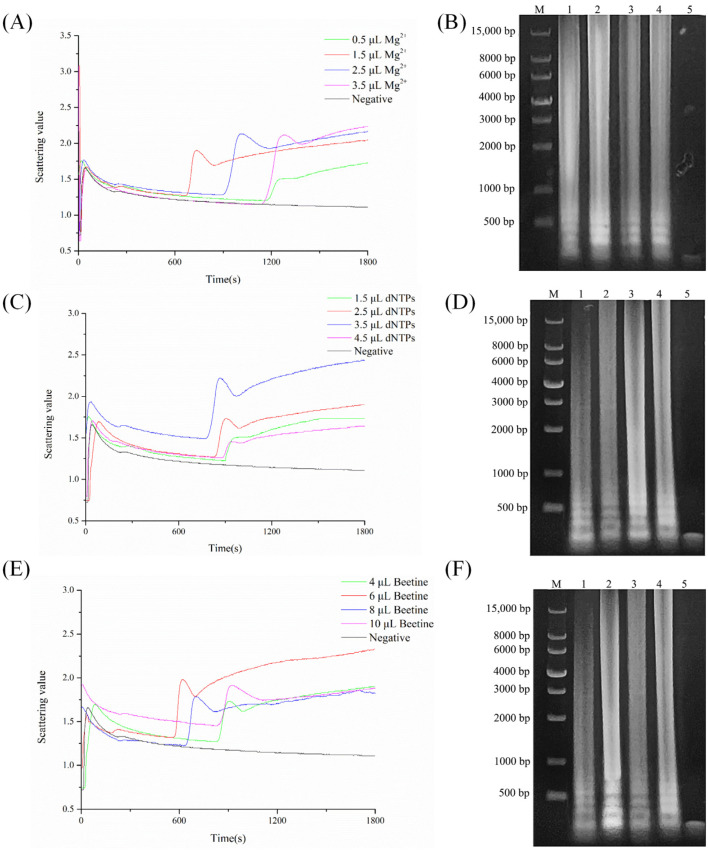
Optimization of different reagents: (**A**) turbidity scatter plots at different volumes of magnesium ion solution; (**B**) electrophoresis of amplification products with M-DNA marker: 1—0.5 µL magnesium ion, 2—1.5 µL magnesium ion, 3—2.5 µL magnesium ion, 4—3.5 µL magnesium ion, 5—negative control; (**C**) turbidity scatter plots at different volumes of dNTP solution. Scattering values and electrophoresis of different volumes of dNTP solution; (**D**) electrophoresis of amplification products with M-DNA marker: 1—1.5 µL dNTPs, 2—2.5 µL dNTPs, 3—3.5 µL dNTPs, 4—4.5 µL dNTPs, 5—negative control; (**E**) turbidity scatter plots at different volumes of betaine solution; (**F**) electrophoresis of amplification products with M-DNA marker: 1—4 µL betaine, 2—6 µL betaine, 3—8 µL betaine, 4—10 µL betaine, 5—negative control.

Magnesium ion is an essential cofactor for polymerases, playing a crucial role in stabilizing nucleotides, amplification systems, and enhancing enzyme activity. Insufficient magnesium ion concentration significantly reduces enzyme activity, while excessive concentrations can catalyze nonspecific amplification. As shown in Figure 3A, under unchanged conditions, varying the amount of magnesium ions resulted in different peak times of turbidity scatter curves. The curve began to peak at 674 s with 1.5 µL of the magnesium ion solution, while peak times were later than 674 s under other conditions. Additionally, from the electrophoresis shown in Figure 3B, lane two (1.5 µL) exhibited the brightest band, whereas lanes one (0.5 µL), three (2.5 µL), and four (3.5 µL) appeared dimmer. Furthermore, lanes three and four showed significantly lower band intensities compared to lanes one and two, likely due to excessive concentration causing nonspecific amplification. Therefore, 1.5 µL of magnesium ions showed optimal reaction efficiency as indicated by increasing byproducts and turbidity during the amplification reaction.

#### 3.1.3. dNTP Amount

An abnormal or failed experiment can result from either excessively high or low dNTP concentrations. As shown in Figure 3C, under unchanged conditions, varying the amount of dNTPs resulted in a peak time of 785 s with 3.5 µL of dNTPs, while peak times were later than 758 s under other conditions, with no scattering values observed in the negative control. Moreover, the curves with dNTP amounts of 1.5 µL and 4.5 µL showed similar peak times, around 900 s, indicating that the optimal dNTP amount ranges from 1.5 µL to 4.5 µL. Hence, 3.5 µL of dNTPs demonstrated the best reaction efficiency. As shown in Figure 3D, under these reaction conditions, lane three exhibited the brightest band, indicating the highest reaction efficiency. Lanes one and two showed very faint bands, indicating lower reaction efficiency. Lane four showed a band intensity similar to lane three but slightly dimmer. Therefore, the optimal amount of dNTPs required for the LAMP turbidity reaction is 3.5 µL.

#### 3.1.4. Betaine Amount

In nucleic acid amplification reactions, betaine plays a crucial role in enhancing DNA amplification efficiency. Its concentration affects enzyme activity; high concentrations can inhibit enzyme activity by interacting with metal ions. As shown in Figure 3E, under unchanged conditions, varying the amount of betaine resulted in a peak time of 569 s with 6 µL of betaine, while peak times were later than 569 s under other conditions, with no significant peak shapes observed in the negative control, indicating failed amplification. Additionally, from Figure 3F, under these reaction conditions, lane two exhibited the brightest band, indicating the highest reaction efficiency. Therefore, the optimal amount of betaine required for the LAMP reaction is 6 µL.

#### 3.1.5. Optimal Reaction System

Through a series of optimizations, the optimal reaction system was determined to be as follows: LAMP Buffer (2.5 µL), betaine (6 µL), magnesium ions (1.5 µL), dNTPs (3.5 µL), BST polymerase (1 µL), primers (2.5 µL), DNA template (2 µL), and ddH_2_O (6 µL) added to the reaction mixture for a total volume of 25 µL.

### 3.2. Specificity of the LAMP Reaction System

**Figure 4:**
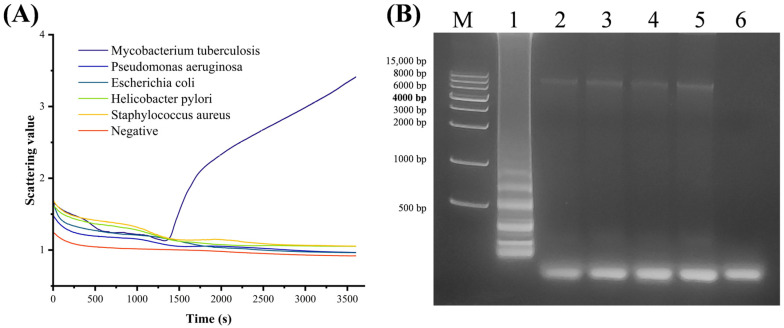
Real-time turbidity LAMP specificity detection and electrophoresis: (**A**) turbidity scatter plots for different kinds of DNA templates; (**B**) electrophoresis of amplification products with M-DNA marker: 1—MTB, 2—*Pseudomonas aeruginosa*, 3—*Escherichia coli*, 4—*Helicobacter pylori*, 5—*Staphylococcus aureus*, 6—negative control.

As shown in Figure 4A, only MTB produced a positive result, with a peak time of approximately 1375 s (23 min) in the scatter curve. Several additional bacteria and the negative control showed no changes in scattering values, indicating no amplification reaction. As shown in Figure 4B, a characteristic LAMP ladder-like band appeared in lane one (MTB), and no amplification bands appeared in the other lanes. Therefore, the three pairs of primers for MTB detection demonstrated good specificity.

### 3.3. Sensitivity of the LAMP Reaction System

**Figure 5:**
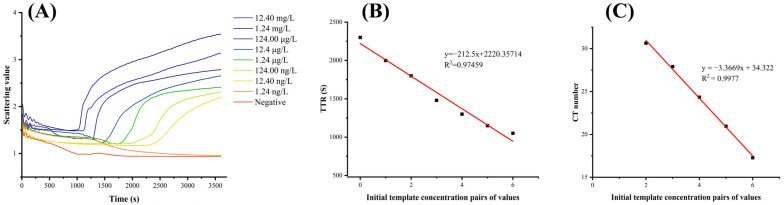
(**A**) Real-time turbidity LAMP sensitivity detection; (**B**) linear relationship between DNA concentration and TTR; (**C**) linear relationship between DNA concentration and CT number.

As shown in Figure 5A, the minimum detection limit of the LAMP turbidity method was 12.40 ng/L (39.58 min). When the DNA concentration was further diluted to 1.24 ng/L, the target nucleic acid could not be detected. Figure 5B shows that the TTR of the DNA template concentration is linearly related to the amplification time, and the R^2^ value is 0.97459. Therefore, the LAMP reaction system for detecting MTB can be used for quantitative analysis. Figure 5C shows that the logarithm of the DNA template concentration is linearly related to the CT. When the template concentration is 12.4 mg/L, the CT value is 17.28 (1050 S) and the TTR is 1044 s, which are very close. When the template concentration is 12.4 µg/L, the CT value is 17.28 (1575 S), and the TTR time is shorter, which is 1480 S. It can be concluded that the results of the LAMP reaction system are within the detection limit, which is equivalent to PCR and can be used for quantitative analysis.

### 3.4. Accuracy of the LAMP Reaction System

**Figure 6:**
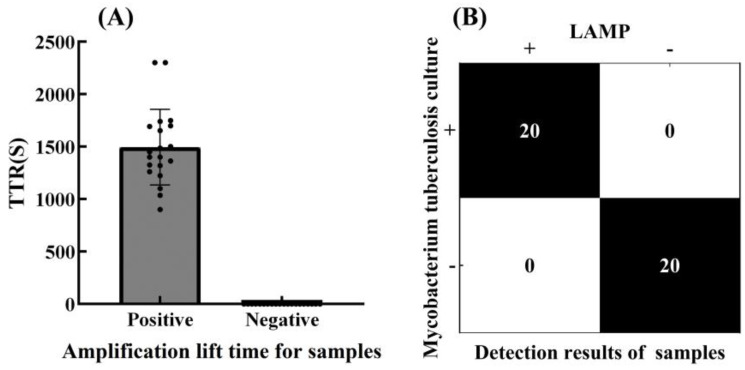
(**A**) Amplification lift time within 60 min for positive and negative samples. The scattered voltage of 20 positive samples reached the threshold value, and 20 negative samples did not reach the threshold value, TTR = 0. (**B**) Results of LAMP and *Mycobacterium tuberculosis* culture for 40 samples. The experimental results were consistent with those of the mycobacterium tuberculosis culture method.

A total of 20 MTB-positive samples and 20 MTB-negative samples were assayed by the self-developed scattering LAMP turbidimeter with the optimal LAMP reaction system. As shown in Figure 6A, the accuracy rate reached 100%, with the amplification lift time for each sample recorded within 60 min based on the threshold value. As shown in Figure 6B, the amplification lift time of the positive samples was 900.4 s–2300 s (24.9 ± 13 min), while no jump was observed for any negative samples. So, the developed LAMP reaction system can be used to efficiently detect authentic MTB with high accuracy and good reproducibility.

## 4. Conclusions

A novel LAMP method based on turbidity scatter for the rapid detection of MTB was established. Three pairs of primers (including loop primers) were designed for the *Ag85B* gene of MTB. The optimal reaction system was determined by investigating the reaction temperature and the amounts of magnesium ions, dNTPs, and betaine. A LAMP turbidity meter was used for nucleic acid amplification detection, avoiding contamination from opening tubes. The detection time was approximately 10–45 min, with a minimum detection limit of 12.40 ng/L and good specificity. The developed LAMP reaction kit and scattering LAMP turbidimeter can be used for the detection of clinical samples. This method provides an efficient, safe, and economical approach for the accurate diagnosis of MTB, supporting decision making for precise prevention and control and thereby maximizing public health benefits.

## Figures and Tables

**Table 1:** Sequence information for *Ag85B* used for LAMP.

Primer Name	Primer Sequence
F3	CTACAAGTGGGAGACGTTCC
B3	ACGGTCCCATCCCTTGG
FIP	GCTACCGGTGGGCTTGACGTAACCAGCGAACTCCCCGA
BIP	GGCATCTCGATGTCCGGCTCCAGCGAACCGGCGTAC
LF	TGCGATTGGCCGACAGC
LB	TGTACCACCCCCAGCAGT

**Table 2:** Optimization of LAMP reaction conditions.

Temperature	T Magnesium Ion Volume	dNTPs Volume	Betaine Volume
(°C)	(µL)	(µL)	(µL)
63	0.5	2.5	4
64	1.5	3.5	6
65	2.5	4.5	8
66	3.5	5.5	10

## Data Availability

The data that support the findings of this study are available from the corresponding authors upon reasonable request.

## Abbreviations

The following abbreviations are used in this manuscript:

TB	Tuberculosis
MTB	*Mycobacterium tuberculosis*
DR-TB	Drug-resistant tuberculosis
TST	Tuberculosis skin test
IGRA	Interferon-γ release assay
BCG	bcg vaccine
LAMP	Loop-mediated isothermal amplification
*Ag85B*	Specific primers for the MTB-specific gene
PCR	Polymerase chain reaction
TTR	Time to Results
TH	Threshold
